# Automated Spatial Brain Normalization and Hindbrain White Matter Reference Tissue Give Improved [^18^F]-Florbetaben PET Quantitation in Alzheimer's Model Mice

**DOI:** 10.3389/fnins.2016.00045

**Published:** 2016-02-29

**Authors:** Felix Overhoff, Matthias Brendel, Anna Jaworska, Viktoria Korzhova, Andreas Delker, Federico Probst, Carola Focke, Franz-Josef Gildehaus, Janette Carlsen, Karlheinz Baumann, Christian Haass, Peter Bartenstein, Jochen Herms, Axel Rominger

**Affiliations:** ^1^Department of Nuclear Medicine, Ludwig-Maximilians-University of MunichMunich, Germany; ^2^DZNE-German Center for Neurodegenerative DiseasesMunich, Germany; ^3^Laboratory of Neurodegeneration, International Institute of Molecular and Cell BiologyWarsaw, Poland; ^4^Roche Pharma Research and Early Development, Neuroscience Discovery, Roche Innovation Center Basel, F. Hoffmann-La Roche LtdBasel, Switzerland; ^5^Munich Cluster for Systems Neurology (SyNergy)Munich, Germany; ^6^Biomedical Center, Ludwig-Maximilians-University of MunichMunich, Germany

**Keywords:** Alzheimer's disease, β-amyloid, [^18^F]-florbetaben, small animal PET, reference region, brain normalization

## Abstract

Preclinical PET studies of β-amyloid (Aβ) accumulation are of growing importance, but comparisons between research sites require standardized and optimized methods for quantitation. Therefore, we aimed to evaluate systematically the (1) impact of an automated algorithm for spatial brain normalization, and (2) intensity scaling methods of different reference regions for Aβ-PET in a large dataset of transgenic mice. PS2APP mice in a 6 week longitudinal setting (*N* = 37) and another set of PS2APP mice at a histologically assessed narrow range of Aβ burden (*N* = 40) were investigated by [^18^F]-florbetaben PET. Manual spatial normalization by three readers at different training levels was performed prior to application of an automated brain spatial normalization and inter-reader agreement was assessed by Fleiss Kappa (κ). For this method the impact of templates at different pathology stages was investigated. Four different reference regions on brain uptake normalization were used to calculate frontal cortical standardized uptake value ratios (SUVR_CTX∕REF_), relative to raw SUV_CTX_. Results were compared on the basis of longitudinal stability (Cohen's d), and in reference to gold standard histopathological quantitation (Pearson's R). Application of an automated brain spatial normalization resulted in nearly perfect agreement (all κ≥0.99) between different readers, with constant or improved correlation with histology. Templates based on inappropriate pathology stage resulted in up to 2.9% systematic bias for SUVR_CTX∕REF_. All SUVR_CTX∕REF_ methods performed better than SUV_CTX_ both with regard to longitudinal stability (*d*≥1.21 vs. *d* = 0.23) and histological gold standard agreement (*R*≥0.66 vs. *R*≥0.31). Voxel-wise analysis suggested a physiologically implausible longitudinal decrease by global mean scaling. The hindbrain white matter reference (*R*_mean_ = 0.75) was slightly superior to the brainstem (*R*_mean_ = 0.74) and the cerebellum (*R*_mean_ = 0.73). Automated brain normalization with reference region templates presents an excellent method to avoid the inter-reader variability in preclinical Aβ-PET scans. Intracerebral reference regions lacking Aβ pathology serve for precise longitudinal *in vivo* quantification of [^18^F]-florbetaben PET. Hindbrain white matter reference performed best when considering the composite of quality criteria.

## Introduction

The steadily growing number of patients suffering from Alzheimer's disease (AD) will place a great burden on healthcare systems in the coming decades, baring development of an effective intervention therapy (Schneider, 2013). Molecular imaging of β-amyloid (Aβ) with positron emission tomography (PET) has given new insight into the progression of AD pathology and has entered clinical diagnostic use (Johnson et al., 2013). Furthermore, PET imaging is increasingly used for detecting cerebral amyloidosis in transgenic mouse models of AD (Manook et al., 2012; Rominger et al., 2013). Small animal PET studies of longitudinal design afford monitoring of the rate of β-amyloid accumulation, and present the possibility of testing interventions for attenuating plaque formation.

Preclinical brain PET imaging frequently suffer from shortcomings such as underpowered study groups, reader dependence of endpoints, varying approaches to data analysis, and inadequate blinding of investigators to treatment groups (Jucker, 2010). Automated template-based normalization of rodent brain to standard coordinates, in analogy to standard methods for analysis of human PET data, has the potential to minimize biases from reader dependence and imperfect blinding, although the reliability of such approaches has yet to be systematically investigated for preclinical Aβ-PET. In particular, the choice of the optimal template for automated spatial normalization of Aβ-PET images may be influenced by pathological features of the particular AD mouse model (Rohlfing et al., 2009). Since this issue has not hitherto been raised in preclinical Aβ-PET imaging, we planned to validate an automated, user-independent approach for spatial normalization by comparing binding results from readers at different training levels before and after normalization, with histopathological examination of fibrillar Aβ as the gold standard. Furthermore, we objected to study the influence of template selection at different pathology stages on the automated spatial normalization of mouse brain Aβ-PET images.

An additional point of contention in preclinical Aβ-PET concerns the method for image intensity scaling, with normalization either to the injected dose, or to tracer uptake in intracerebral reference regions. Just as in human PET studies with Aβ-PET tracer, the choice of reference regions is crucial for preclinical models, which have characteristic patterns of Aβ deposition. In human Aβ-PET imaging, the whole cerebellum and the cerebellar gray matter have been the preferred reference regions for most large scale quantitative investigations (Vandenberghe et al., 2010; Barthel and Sabri, 2011; Clark et al., 2011). However, recent human studies with fluorinated amyloid tracers have revealed longitudinal stabilization of standardized uptake value ratios (SUVR), with use of white matter or brain stem reference regions, this despite the ongoing accumulation of Aβ plaques as confirmed by histology (Landau et al., 2014, 2015; Brendel et al., 2015a; Chen et al., 2015). There has so far been no systematic comparison of scaling methods for mouse Aβ-PET imaging. Consequently, we aimed to compare different scaling methods and reference regions in a large series of Aβ studies with [^18^F]-florbetaben (FBB) in an established AD mouse model, with regard to accuracy of the PET method in capturing longitudinal changes in Aβ-deposition, with terminal histological plaque quantitation serving as the gold standard. The overall aims of this study were to investigate if automated brain spatial normalization is beneficial for quantitation of preclinical Aβ-PET, and to identify an optimally performing method for intensity scaling.

## Materials and methods

### Animal model and study design

All experiments were performed in compliance with the National Guidelines for Animal Protection, Germany, with approval of the local animal care committee of the Government of Oberbayern (Regierung Oberbayern), and overseen by a veterinarian. Anesthesia was performed with isoflurane 1.5%. Mice were killed by cervical dislocation in a state of deep narcosis.

The transgenic B6.PS2APP (line B6.152H) is homozygous for both the human presenilin (PS) 2, N141I mutation and the human amyloid precursor protein (APP) K670N, M671L mutation. The APP and PS2 transgenes are driven by mouse Thy-1 and mouse prion promoters, respectively. This line had been created by co-injection of both transgenes into C57Bl/6 zygotes (Richards et al., 2003). Homozygous B6.PS2APP mice show first appearance of plaques in the cortex and hippocampus at 5–6 months of age (Ozmen et al., 2009).

Aβ-PET scans (*N* = 114) were used from two studies wherein drug-naive longitudinal FBB-PET recordings were obtained in PS2APP mice (*N* = 37) at a short interval (8 and 9.5 months of age). Furthermore, we also used terminal Aβ-PET recordings (*N* = 40; range: 13–16 months; *N* = 24 from the longitudinal set and *N* = 16 from another historical investigation), in which histologically quantified terminal plaque load was measured. We defined the different ages for Aβ-PET imaging as “BL” for the baseline at 8 months (*N* = 37 PS2APP mice), “FU” for the follow-up of the same group at 9.5 months (*N* = 37 PS2APP mice), and “TER” for the terminal Aβ-PET recordings obtained between 13 and 16 months of age (*N* = 40 PS2APP mice).

### Radiochemistry

The [^18^F]-florbetaben precursor (Piramal Imaging, Berlin) was radiolabeled by the method of Zhang et al. (2005), with slight modifications. This procedure is described elsewhere (Rominger et al., 2013), and yields a radiochemical purity exceeding 98% and specific activity of 50–90 GBq/μmol at the end of synthesis.

### Amyloid pet acquisition and reconstruction

Image acquisition and reconstruction followed a standardized protocol (Brendel et al., 2015b). Mice were anesthetized with isoflurane (1.5%, delivered via a mask at 3.5 L/min in oxygen) and received bolus injection of 10.1 ± 2.3 MBq of [^18^F]-florbetaben in 150 μL of saline to a tail vein. Following placement in the tomograph (Siemens Inveon DPET), a single frame emission recording for the interval 30–60 min p.i. followed by a 15 min transmission scan was obtained using a rotating [^57^Co] point source. The image reconstruction procedure consisted of an three-dimensional ordered subset expectation maximization (OSEM) with four iterations and 12 subsets followed by a maximum *a posteriori* (MAP) algorithm with 32 iterations. Scatter and attenuation correction were performed and a decay correction for [^18^F] was applied. With a zoom factor of 1.0 and a128 × 128 × 159 matrix, a final voxel dimension of 0.78 × 0.78 × 0.80 mm was obtained.

### Image analysis

#### Spatial normalization

Aβ-PET images were first blinded to the reader by coding of the Aβ-PET files. Images were coregistered to a 3T magnetic resonance imaging (MRI) mouse brain template (Dorr et al., 2007) by a rigid manual fusion, using the PMOD FUSION tool (v. 3.4 PMOD Technologies, Zürich). The spatial normalization was independently performed by an expert (>1000 fusions), an experienced reader (~150 fusions) as well as a novice reader who had been trained for 4 h over the course of 2 days. The expert fusion was repeated to assess test-retest variability (%) for operators with high training level.

**Figure 1:**
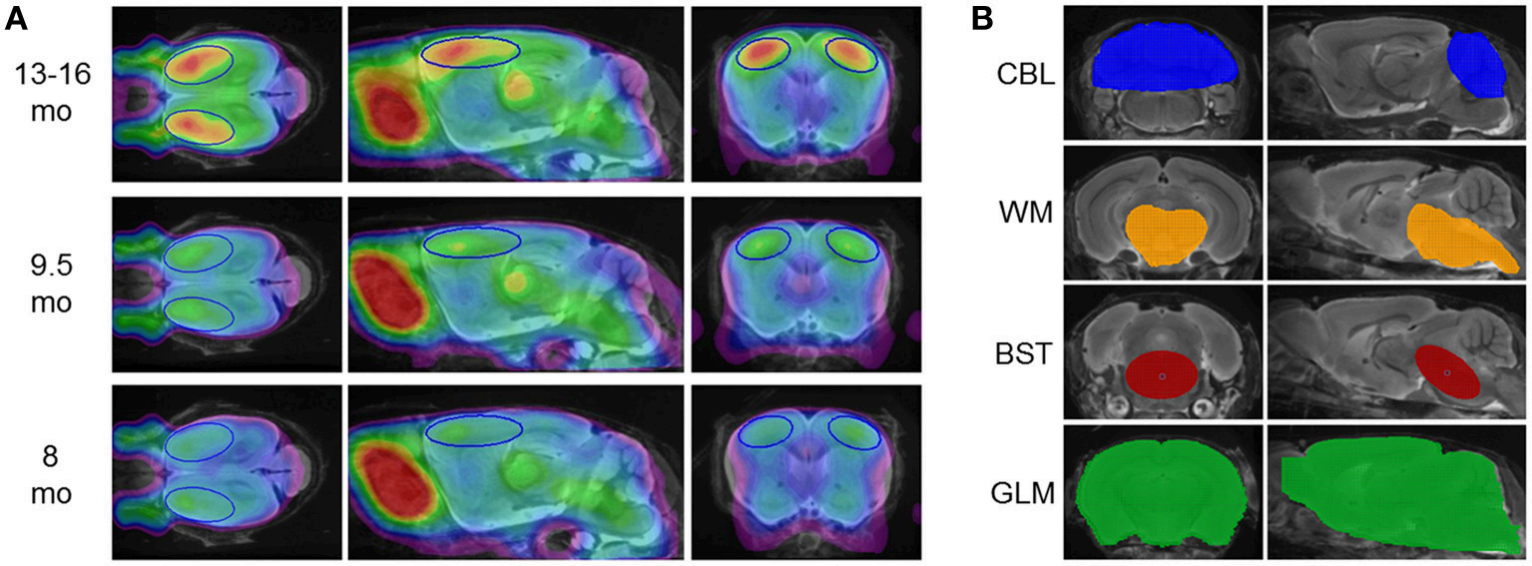
**(A)** [^18^F]-florbetaben PET templates at different pathology stages, deriving from mean findings in groups of PS2APP mice aged 8, 9.5, and 13–16 months. PET images are superimposed on an MRI-based mouse brain atlas (Dorr et al., 2007) for anatomical reference. The frontal cortical target VOI is depicted in blue. **(B)** Reference region VOIs are illustrated on the same MRI mouse atlas; from top to bottom: cerebellum (blue; CBL), hindbrain white matter (orange; WM), brainstem (red; BST), whole brain (green; GLM).

All spatial normalized images of the expert reader were intensity scaled to the injected dose and multiplied by the individual body weight to give standardized uptake value (SUV) images. Then age-dependent groups (*N* = 37 or *N* = 40 images each) from PS2APP mice were averaged for the generation of three different templates defined at increasing stages of amyloid pathology (Figure 1A).

Subsequently, fused images from all readers were spatially normalized on all different templates by applying an automated nonlinear transformation, which removed the main differences in brain size and shape using the normalization algorithm in SPM5 (Wellcome Department of Cognitive Neurology, London, UK) implemented within the PMOD FUSION tool (V. 3.4 PMOD Technologies, Zürich). This algorithm was set to mouse brain dimensions using standardized settings (equal modality; nonlinear warping; 16 iterations; frequency cutoff 3; regularization 1.0; no thresholding). The procedure was tested with and without transient input smoothing by applying a 0.6 mm Gaussian to the image before initiation of warping operations. The calculated transformation matrix was saved and finally applied to the “raw” input image to avoid loss of resolution due to repeated image resampling.

#### Inter-reader analyses

For inter-reader analyses, Fleiss Kappa (κ) was calculated for TER results (SUV_CTX_ and SUVR_CTX∕REF_ as described below) from expert vs. experienced, experienced vs. novice, expert vs. novice, both before and after automated brain normalization. Furthermore, inter-reader variability (%) was calculated, and compared to the expert test-retest variability (%). For statistical testing of absolute differences for SUV_CTX_ and SUVR_CTX∕REF_ between different readers before and after brain normalization a permutation test was performed as described in Section Statistics below and was also used to test for significant differences between inter-reader variability (%) and expert test-retest variability (%).

#### Analysis of template influence on automated brain intensity normalization

To assess the impact of stage specific templates, TER results of the expert before brain normalization were compared with TER results after brain normalization to TER, FU, and BL templates by calculation of error-(%), with findings of the expert considered as the standard of truth. A paired *t*-test was used for statistical testing of significant alterations in SUV_CTX_ and SUVR_CTX∕REF_ (as described below) resulting from different templates.

#### Scaling

A total of five intensity scaling methods were performed, all using VOIs predefined in the final image space, which was identical for MRI and [^18^F]-florbetaben templates. Beside calculation of frontal cortical SUV_CTX_, we tested intensity scaling by four different reference regions (Figure 1B):

1. Whole cerebellum (CBL) with a volume of 65 mm^3^.
2. Hindbrain white matter (WM), including high unspecific tracer retention areas (threshold-based) such as pons, midbrain, cerebellar peduncle, cranial hypothalamus and caudal thalamus, with a total volume of 67 mm^3^.
3. Brainstem (BST), an oval shaped region extending from pons to midbrain, with a volume of 24 mm^3^.
4. Whole brain as the global mean (GLM), with a volume of 525 mm^3^.

As target regions two bilateral frontal cortex VOIs comprising 12 mm^3^ each (Figure 1A), were employed for calculation of [^18^F]-florbetaben cortex-to-reference SUVR_CTX∕REF_.

##### Longitudinal stability

Scaling methods were tested against each other by evaluating the longitudinal stability of the endpoint (SUV_CTX_ or SUVR_CTX∕REF_) over the brief 6 week interval from BL to FU in *N* = 37 animals. Pearson's coefficients of correlation (R) were calculated for SUV_CTX_ and SUVR_CTX∕REF_ values of the four different reference regions, given the assumption that the mouse model is characterized by a nearly linear progression of amyloidosis over time, as supported by findings from our previous study (Brendel et al., 2015b). The variance of BL and FU groups, expressed by SD-(%), was calculated as an indicator of intra-group stability. Effect sizes (Cohen's d) for the resulting differences between the two sequential Aβ-PET scans were calculated as an additional quality criterion in the longitudinal design.

##### Longitudinal regional analyses

To test the impact of different scaling methods for Aβ-PET on the detected differences in longitudinal data independently from the cortical target VOI, we assessed alterations in FBB-binding between BL and FU voxel-wise by statistical parametric mapping (SPM). We used SPM5 routines implemented in MATLAB (version 7.1), adapted from Sawiak et al. (2009) for mouse data. For SUV_CTX_ and each reference region approach, we performed a paired *t*-test for Aβ-PET images (FU vs. BL) of PS2APP (*N* = 37) mice, and thus assessed increases or decreases over 6 weeks of follow up.

### Histochemical analyses

Histochemical analyses were performed in a matching frontal cortex region of interest as the gold standard of amyloid burden, for evaluating reliability of frontal cortical SUV_CTX_ and four different SUVR_CTX∕REF_ results. The procedure followed a standardized protocol wherein cortical plaque load (%) was calculated for each animal (Brendel et al., 2015b). For correlation analyses of the terminal Aβ-PET estimates (*N* = 40) with plaque load (%), Pearson's coefficients of correlation (R) were calculated with and without brain normalization and for all different intensity normalization methods. Significant differences between correlation coefficients before and after spatial brain normalization, between different readers, and between different intensity normalization methods were assessed by an extended Fisher's transformation approach as described in Section Statistics.

### Statistics

A permutation test was used to test for significance of not normally distributed differences between two readers before and after normalization and for the comparison of inter-reader variability (%) with test-retest variability (%) of the expert. Absolute values were used for these comparisons. For permutation testing, the results were pooled and a loop rearranging the pooled results into two groups (with 1 million repeats) was coded within Matlab 7.12.0. The originally observed results of difference between manually acquired and normalized data or of difference between test-retest expert variability (%) and inter-reader variability (%) were defined as target values. For every resampled pair the mean result was calculated and each mean result equal to or higher than the target value was counted automatically. Finally, the total count was set in relation to the number of repeats to obtain the *p*-value.

Significant differences between two dependent correlations with one variable in common (plaque load %) were assessed by an extended Fisher's transformation approach (Lee and Preacher, 2013). First, each correlation coefficient was converted into a z-score using Fisher's r-to-z transformation. Then, asymptotic covariance of the estimates was computed. Finally, these quantities were used in an asymptotic *z*-test. A threshold of *p* < 0.05 was considered to be significant for rejection of the null hypothesis in all statistical tests.

## Results

### Spatial normalization

#### Automated brain normalization significantly reduces inter-reader variability

**Figure 2:**
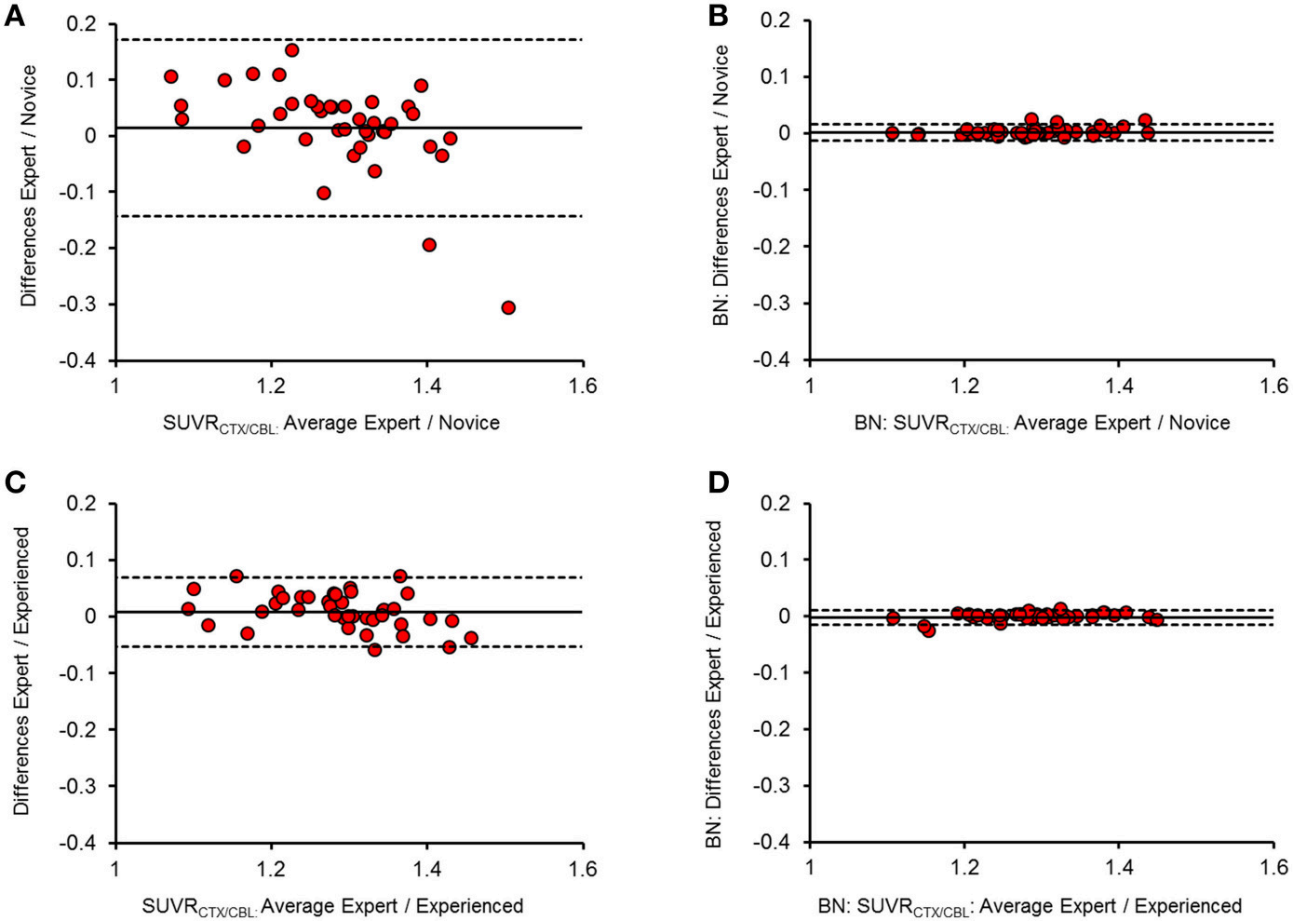
**Bland-Altman plots of the inter-reader SUVR_**CTX∕CBL**_(***n*** = 40) agreement before (A,C) and after (B,D) brain spatial normalization**. Difference (y-axis) and mean (x-axis) between two readers are illustrated against each other, with the mean difference presented by the thick line and the upper (+1.96 SDs) and lower limits (−1.96 SDs) of agreement by the dashed lines. Panels **(A,B)** show the comparison between expert and novice reader, while **(C,D)** show the relation between expert and experienced reader.

The intra-reader test-retest variability of SUVR_CTX∕REF_ for the expert reader was 1.4 ± 1.0% (range: 0.9–2.9% for different reference regions). Inter-reader agreement for SUVR_CTX∕REF_ was very high between the expert and the experienced reader without brain pre-normalization (κ = 0.97 ± 0.02; inter-reader variability 1.4 ± 0.8%), indicating a very high reproducibility when both readers are at a high training level without significant differences when compared to the expert test-retest variability (all *p* = n.s.). However, lower inter-reader agreement including some strong outliers occurred between novice reader and expert (κ = 0.89 ± 0.08; inter-reader variability 3.0 ± 1.8%; all *p* < 0.01), or novice and experienced reader (κ = 0.88 ± 0.06; inter-reader variability 2.9 ± 1.5%; all *p* < 0.01). Especially the agreement for SUVR_CTX∕CBL_ was lower in the latter two contrasts (κ = 0.77∕0.79; inter-reader variability = 5.6%/5.2%) when only the manual PET-MRI fusions were compared. After automated spatial brain normalization, all κ values were ≥ 0.99 and the maximal inter-reader variability was 0.6%. All differences between readers were significantly lowered by the automated brain normalization procedure (*p* < 0.001 for all reference regions). Brain normalization without transient input smoothing (0.6 mm) during the automated coregistration process led to some instances of strong image distortions, and was therefore dismissed. Representative approximations of SUVR_CTX∕CBL_ between readers after brain spatial normalization are illustrated in Bland-Altman Plots (Figure 2) and all κ-values/%-variabilities are reported in Table 1.

**Table 1:** **Inter-reader agreement**.

**κ/inter-reader variability (%)**				**Test-retest variability**
**Reference**	**Expert-experienced (κ/%)**	**Experienced-novice (κ/%)**	**Expert-novice (κ/%)**	**Expert (%)**
**RAW FUSION**				
CBL	0.95/2.6	0.79/5.2^*^	0.77/5.6^*^	2.9
WM	0.98/1.0	0.91/2.4^**^	0.93/2.1^**^	0.9
BST	0.99/0.9	0.94/2.0^**^	0.96/1.8^**^	0.9
GLM	0.95/1.2	0.88/2.1^**^	0.89/2.3^**^	0.9
MEAN ± SD	0.97 ± 0.02/1.4 ± 0.8	0.88 ± 0.06/2.9 ± 1.5^**^	0.89 ± 0.08/3.0 ± 1.8^**^	1.4 ± 1.0
**BRAIN NORMALIZATION**				
CBL	1.00/0.5	0.99/0.6	1.00/0.5	
WM	1.00/0.2	0.99/0.4	0.99/0.4	
BST	1.00/0.2	0.99/0.4	0.99/0.5	
GLM	1.00/0.2	0.99/0.5	0.99/0.4	
MEAN ± SD	1.00 ± 0.00/0.2 ± 0.1	0.99 ± 0.00/0.5 ± 0.1	0.99 ± 0.00/0.5 ± 0.1	

Inter-reader agreement expressed by Fleiss κ and inter-reader variability (%) before and after brain normalization (BN) for all different reference regions. For the raw fusion the test-retest variability of the expert is given.

* p < 0.01;

** *p < 0.001 for interreader-variability (%) vs. expert test-retest variability (%), permutation test*.

#### Template characteristics influence brain normalization results

**Figure 3:**
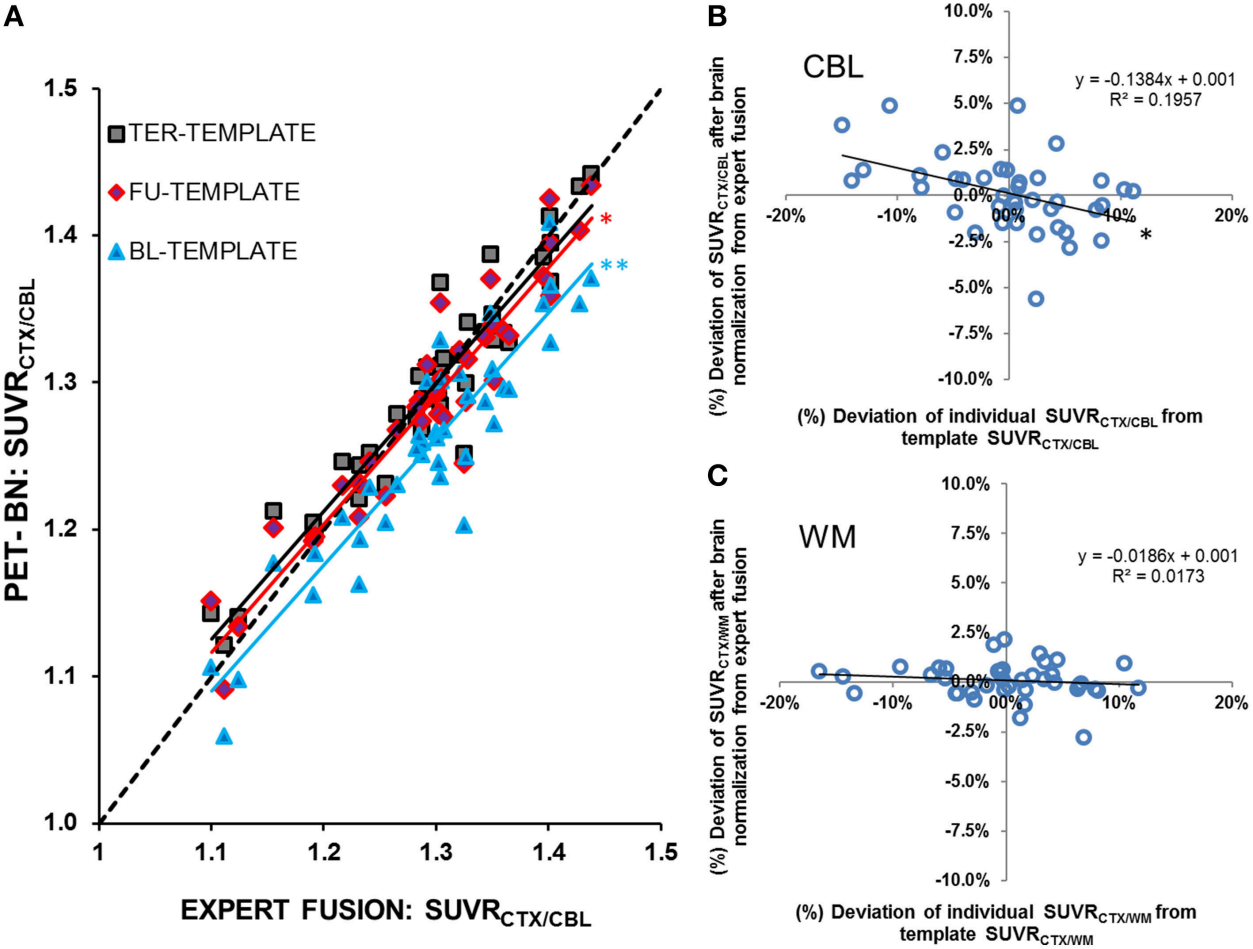
**(A)** SUVR_CTX∕CBL_ from *N* = 40 PS2APP mice aged 13–16 months after brain spatial normalization to a stage-specific (high) tracer uptake template (TER-TEMPLATE, black-gray squares), an intermediate tracer uptake template (FU-TEMPLATE, red-purple diamonds) and a low tracer uptake template (BL-TEMPLATE, blue triangles). Lower uptake templates gave systematic underestimation of SUVR_CTX∕CBL_after automated brain normalization when compared to expert fusion. With the stage-specific template, minor underestimations were observed for high SUVR_CTX∕CBL_ and *vice versa*. Dashed line represents the line of perfect identity (*R* = 1) between raw expert fusion and brain normalization). Significant differences (paired *t*-test) resulting from template mismatches vs. the stage specific TER-template are indicated by ^*^p < 0.05; ^**^p < 0.001. Relationships between (%)-deviations of individual SUVR_CTX∕REF_ from the template SUVR_CTX∕REF_ and the (%)-deviation of SUVR_CTX∕REF_ from the expert manual fusion after the brain normalization are illustrated for **(B)** the cerebellar and **(C)** the hindbrain white matter reference region. Significant correlation is indicated by ^*^p < 0.01.

FBB uptake in the TER scans was underestimated when spatially normalized to templates from BL and FU disease stages, under the assumption that the expert manual fusion serves as the standard of truth. This effect was most obvious with the cerebellum serving as the reference region (BL-template: −0.6 ± 2.1%, *p* < 0.05/FU-template: −2.9 ± 2.4%, *p* < 0.001; Figure 3A). With the stage specific TER template, low SUVR_CTX∕REF_ values tended to be overestimated, whereas high results were underestimated, but the mean error-(%) was only ±0.2%. Based on these findings, we attempted to quantify the bias deriving from the deviation of individual SUVR_CTX∕REF_ from the template SUVR_CTX∕REF_ by constructing a function defining the relationship between error-(%) and the deviation-(%) = (individual - SUVR_CTX∕REF_-template-SUVR_CTX∕REF_) / template - SUVR_CTX∕REF^*^_100. Using this function we estimated a bias of ±13.8% (linear regression: *R* = −0.44;*p* < 0.01) for SUVR_CTX∕*CBL*_(Figure 3B), meaning that an individual mouse with a 10% higher SUVR_CTX∕CBL_ compared to the template results gives on average a 1.38% lower result after brain intensity normalization. Respective bias was similarly high for SUVR_CTX∕GLM_ (±10.1%; linear regression: *R* = −0.34;*p < 0.05*), but distinctly lower for SUVR_CTX∕WM_ (±1.9%; linear regression: *R* = −0.13; *p* = n.s.; Figure 3C) and SUVR_CTX∕BST_ (±1.7%; linear regression: *R* = −0.16; *p* = n.s.). Absolute biases in SUVR_CTX∕CBL_ and SUVR_CTX∕GLM_ were significantly higher when compared to SUVR_CTX∕WM_ or SUVR_CTX∕BST_ (all *p* < 0.001).

### Longitudinal analysis

#### Intracerebral reference regions give lower variance and higher longitudinal effect sizes when compared to SUV

Implausibly low longitudinal increases of FBB binding were obtained through generation of ordinary SUV_CTX_ estimates (*R* = −0.12, *p* = n.s.), which is explicable by the rather high relative standard deviations in the BL (15.9%) and FU (13.2%) groups. Each of the four intracerebral reference regions stabilized the Aβ-PET SUVR_CTX∕REF_ estimates, and resulted in distinctly lower variance (2.3–4.6%), with highest agreement between BL and FU for SUVR_CTX∕WM_ (*R* = 0.64, *p* < 0.001). Highest effect sizes for the age-dependent increases were found with SUVR_CTX∕WM_ and SUVR_CTX∕BST_ (*d* = 1.64), which exceeded that for SUVR_CTX∕CBL_ (*d* = 1.23) and SUVR_CTX∕GLM_ (*d* = 1.21). Details of this analysis are provided in Table 2.

**Table 2:** **Longitudinal 6-week follow-up**.

	**BL MEAN**	**BL SD (%)**	**FU MEAN**	**FU SD (%)**	***d***	***R***
SUV_CTX_	0.48	0.08 (15.9%)	0.50	0.07 (13.2%)	0.23	−0.12
SUVR_CTX∕CBL_	1.10	0.04 (3.6%)	1.16	0.05 (4.4%)	1.23	0.51
SUVR_CTX∕WM_	0.92	0.03 (3.1%)	0.98	0.04 (4.1%)	1.64	0.64
SUVR_CTX∕BST_	0.90	0.03 (3.6%)	0.96	0.04 (4.6%)	1.64	0.60
SUVR_CTX∕GLM_	1.06	0.02 (2.3%)	1.10	0.04 (3.4%)	1.21	0.63

*Results from the longitudinal 6 week follow-up in (N = 37) PS2APP mice. Mean cortical FBB PET values and their SD are given for BL and FU scans for the different scaling methods together with the Cohen's effect size (d) and BL-FU correlation (R)*.

#### Global mean scaling gives physiologically implausible longitudinal intensity decreases in the hindbrain

**Figure 4:**
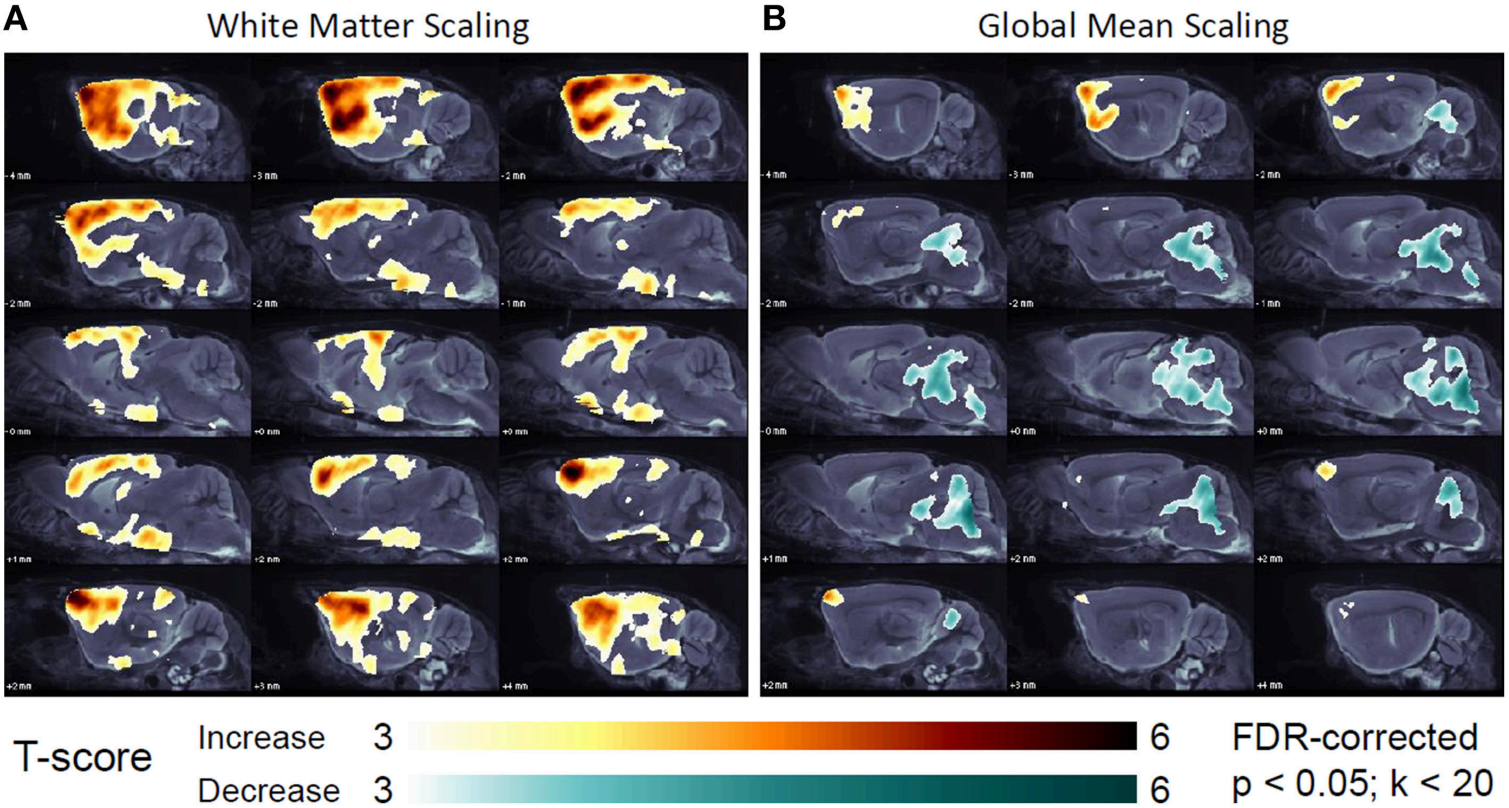
Longitudinal changes in regional [^18^F]-florbetaben uptake in the short-term 6 week follow-up, study of (*N* = 37) PS2APP mice after intensity scaling to the hindbrain white matter **(A)** and the global mean uptake **(B)** as assessed by SPM (FDR-corrected; *p* < 0.05; *k* < 20). T-contrasts expressing significant longitudinal increases (yellow-red) and decreases (turquois-green) are projected upon sagittal slices of the MRI mouse brain atlas.

Voxel-wise analyses revealed a longitudinal progression of amyloidosis from BL to FU in the forebrain for all reference regions in 648,691 voxels (CBL), 662,374 voxels (WM), 612,517 voxels (BST), and 47,247 voxel (GLM) (all FDR-corrected; *p* < 0.05; Figure 4A). Together with the distinctly lower number voxels with temporally increasing SUVR to GLM scaling, 156,066 voxels in the hindbrain (pons, midbrain, and cerebellum) indicated an implausible decrease for SUVR from BL to FU (FDR-corrected; *p* < 0.05; Figure 4B). No amyloid or vascular pathology is known in these regions in PS2APP mice, nor were there any plausible physiological or pathophysiological explanations for a marked apparent decrease in the relative decrease in the hindbrain Aβ-PET signal in a 6 weeks' follow-up. No significantly changing voxels were found when performing SPM with raw SUV images.

### Intensity scaling by intracerebral reference regions gives superior agreement with histology compared to SUV, and is further improved by automated brain spatial normalization

**Figure 5:**
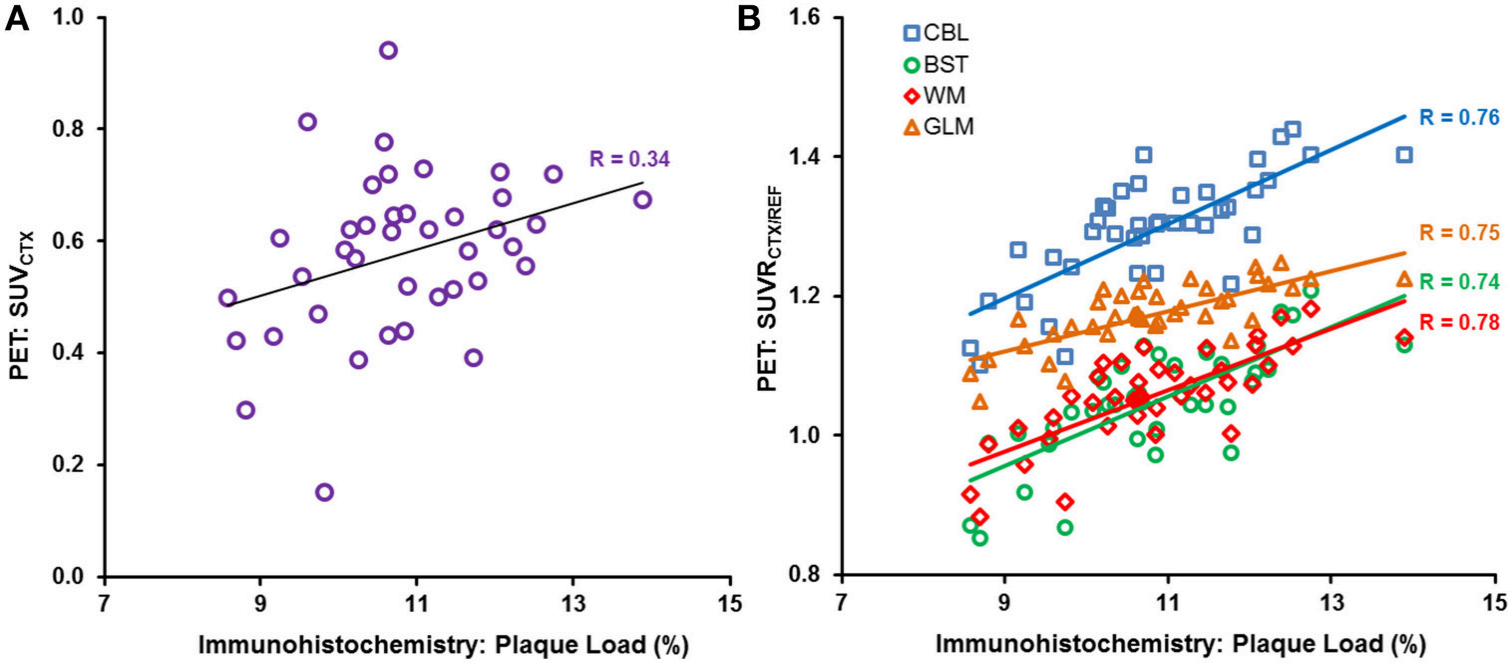
Individual **(A)** SUV_CTX_ and **(B)** SUVR_CTX∕REF_ PET correlations (expert manual fusion) with gold standard plaque load (%), as assessed by histochemistry, in (*N* = 40) PS2APP mice aged 13–16 months, are illustrated for SUV_CTX_ (purple circles) and the different reference regions, i.e., cerebellum (CBL, blue squares), hindbrain white matter (WM, red diamonds), brain stem (BST, green circles), and global mean (GLM, orange triangle).

The cortical plaque load (%) in *N* = 40 PS2APP aged 13–16 months mice was 10.8 ± 1.2%. The correlation between Aβ-PET and plaque load (%) was poor (*R* ≤ 0.34, *p* < 0.05) for the case of plain SUV_CTX_ (Figure 5A), and consistent significantly lower when compared to any intensity scaling to intracerebral reference regions (all *p* < 0.001). SUVR_CTX∕REF_ estimates (Figure 5B) revealed a high correlation (*R*≥0.66, *p* < 0.001) with plaque load (%) in this homogeneous sample of PS2APP mice regardless of the operator training status, or application of automated brain spatial normalization. Without automated brain normalization, the correlation to histology of the manual coregistration by the novice reader was significantly lower (*R*_max_ = 0.70) when compared to the expert (*R*_max_ = 0.78; *p* < 0.01) or experienced reader (*R*_max_ = 0.76; *p* < 0.05). Application of spatial brain normalization harmonized this correlation between different readers (*R*_max_ = 0.75–0.76; no significant differences between readers). In this regard, manual coregistration by the novice reader was significantly improved by automated brain normalization (all reference regions: *p* < 0.05), whereas the changes for the expert and experienced reader did not show any significant alterations (all reference regions: *p* = n.s.). Scaling to WM or GLM (*R*_mean_ = 0.75) was slightly superior (*p* = n.s.) to BST (*R*_mean_ = 0.74) or CBL (*R*_mean_ = 0.73). All correlations are provided in Table 3.

**Table 3:** **Histology agreement**.

**Reader**	**Expert**		**Experienced**		**Novice**	
**RAW/BN**	**RAW**	**BN**	**RAW**	**BN**	**RAW**	**BN**
SUV_CTX_	0.34^**^	0.34^**^	0.34^**^	0.34^**^	0.32^**^	0.31^**^
SUVR_CTX∕CBL_	0.76	0.75	0.76	0.74	0.66^*^	0.74
SUVR_CTX∕WM_	0.78	0.76	0.76	0.76	0.69^*^	0.75
SUVR_CTX∕BST_	0.74	0.74	0.74	0.73	0.70^*^	0.73
SUVR_CTX∕GLM_	0.75	0.76	0.76	0.76	0.70^*^	0.75

Correlations (R) with plaque load (%) as assessed by histochemistry for expert, experienced and novice reader for the different scaling methods before (RAW) and after brain normalization (BN). Significant differences between correlation coefficients are indicated for SUV_CTX_ vs. any SUVR_CTX∕REF_ and for raw fusions vs. brain normalizations of the same reader

* p < 0.05;

** *p < 0.001*.

### Best global performance is archived by hindbrain white matter intensity scaling

Based on the different analyses (brain normalization, group analysis, longitudinal assessment, and correlation with histology as the gold standard) we defined five categories, which need to be addressed when considering the optimal intensity scaling for [^18^F]-florbetaben Aβ-PET imaging in mice (Table 4). (1) The impact of single animal results analyzed by templates used for automated brain normalization should be minimal, but this was not the case for cerebellar and global mean intensity scaling. (2) Variance in groups of mice should not be artifactually raised by the method, which was the case for raw SUV_CTX_. (3) The effect sizes regarding longitudinal progression of amyloidosis in the frontal cortical target region should be captured as best possible, which was the case for hindbrain white matter and brainstem intensity scalings. (4) Longitudinal results should not be affected by presence of amyloid pathology in the reference region, which was the case for the global mean intensity scaling. (5) Aβ-PET signal in single animals should correlate as best possible with the histological gold standard, which was the case for all intracerebral reference regions. There was trend toward better agreement for hindbrain white matter and global mean intensity scaling, compared to cerebellum or brainstem scaling. Thus, in summary the hindbrain white matter scaling performed best when considering the composite of factors.

**Table 4:** **Intensity scaling summary**.

	**Template affection**	**Variance**	**Longitudinal effect size**	**Pathology affection**	**Histology**
SUV_CTX_	o	−	−	o	−
SUVR_CTX∕CBL_	−	+	+	o	+
SUVR_CTX∕WM_	o	+	++	o	+(+)
SUVR_CTX∕BST_	o	+	++	o	+
SUVR_CTX∕GLM_	−	+	+	−	+(+)

*Significant advantages (moderate: +, light green; large: ++, dark green)/disadvantages (−, light red) of different SUV_CTX_ and SUVR calculations are illustrated in five categories, which are deemed relevant for analysis of [^18^F]-florbetaben measurements in the mouse brain. “o” indicate neutral condition; bracketed “+” indicate a trend to advantage. “Template affection” refers to results Section Template Characteristics Influence Brain Normalization Results. “Variance” refers to result Section Intracerebral Reference Regions give Lower Variance and Higher Longitudinal Effect Sizes When Compared to SUV. “Longitudinal effect size” refers to result Section Intracerebral Reference Regions Give Lower Variance and Higher Longitudinal Effect Sizes When Compared to SUV. “Pathology affection” refers to result Section Global Mean Scaling Gives Physiologically Implausible Longitudinal Intensity Decreases in the Hindbrain. “Histology” refers to result Section Intensity Scaling by Intracerebral Reference Regions Gives Superior Agreement with Histology Compared to SUV, and is Further Improved by Automated Brain Spatial Normalization*.

## Discussion

We present a large-scale evaluation of automated spatial brain normalization and a systematic comparison of different intensity scaling methods for preclinical Aβ-PET imaging with FBB. Our established Aβ-PET methodologies were challenged by investigation of a homogeneous PS2APP data set, including a short-term longitudinal follow-up, and histologically validated gold standard results, all characterized by a rather narrow range of plaque burden; this bodes well for sensitive detection of treatment effects. Automated spatial brain normalization reliably harmonized the results of different readers even if poorly trained, while the correspondence with histological gold standard assessments remained stable or even improved. Scaling by intracerebral reference regions (SUVR_CTX∕REF_) was distinctly superior to plain SUV_CTX_, both with regard to longitudinal stabilization and agreement with histology. Global mean intensity scaling revealed a comparable correlation with histology when contrasted to reference regions demonstrably devoid of Aβ pathology, but this procedure impaired detection of longitudinal increases in Aβ in cortex, and returned physiologically implausible decreases in brain regions lacking Aβ burden. A hindbrain white matter reference was slightly superior to the cerebellar reference due, we suppose, to lesser bias from bone uptake and template differences.

### Automated brain normalization

While small animal Aβ-PET studies experienced growing interest in the recent years the methodological tools used for their interpretation were rather heterogeneous. Spatial normalization has hitherto mostly been done on MRI templates (Poisnel et al., 2012; von Reutern et al., 2013), or with a hybrid PET-CT apparatus (Snellman et al., 2013). One previous investigation of this type used SPM-based automated brain normalization (Rojas et al., 2013), and in most studies, the tracer uptake was scaled to a cerebellum reference region (Maeda et al., 2007; Kuntner et al., 2009; Manook et al., 2012; Poisnel et al., 2012; Snellman et al., 2013), although some have employed plain SUV for their analyses (Waldron et al., 2015). However, with respect to the small effect sizes to brief follow-up in AD mice, and the susceptibility of our SUV_CTX_ results to methodological inaccuracies, it is crucial that Aβ-PET data be analyzed with robust, reliable and reader-independent strategies, so as to ensure accuracy of the study outcomes.

To our knowledge there have not been any studies systematically investigating the inter-reader variability as a limitation of small animal Aβ-PET studies, or indeed for any other classes of radioligand. Our investigations were able to show that manual coregistration of Aβ-PET images contributes substantially to variation of results between readers, especially when they have less experience. A further disadvantage of a manual approach arises from the blinding which is necessary for preclinical drug trials (Jucker, 2010). Thus, an experienced reader is usually able to visually identify animals with different extent of the Aβ pathology. So even if differing groups are blinded to the reader, the visual impressions of the images potentially influence the reader. The automated brain normalization diminishes the impact of these influences toward zero as SUVR_CTX∕REF_ values show nearly perfect inter-reader agreement after processing as inter-reader differences were significantly lowered by the method (Figure 2). Thus, the brain normalization brings about a pseudo-blinding for small animal Aβ-PET analysis, and guarantees minimization of inter-reader variance.

While aiming to improve the inter-reader agreement, the automated brain normalization method should not reduce the ultimate correlation between the PET endpoint with the histological gold standard of amyloid burden. Our results clearly indicate that correlation coefficients of PET results with plaque burden remain equal after brain spatial normalization for well-trained readers, whereas the agreement between PET and histology is significantly improved for less trained operators (Table 3): this is an important precondition for stable results in longitudinal or multicentre studies.

We also observed an effect of applying Aβ-PET templates at different pathology stages. Although this effect was never more than 2.9% of the group mean, this avoidable systematic error could potentially bias Aβ-PET outcomes in treatment studies with small effect sizes (Balducci et al., 2014; Brendel et al., 2015c). This bias of inappropriate templates is unsurprising, as automated brain normalization orientates mainly at edges and high uptake regions of the image (Gispert et al., 2003). High plaque burden in the forebrain results in a cortical hot-spot and sharper cortex-to-extracortical contrasts for Aβ-PET mouse brain images, which influences the quality of spatial normalization when this hot region is absent from the utilized template or *vice versa*. In practice, this mismatch tends to shrink the scaled PET image, leading to underestimation in mice with higher plaque burden than in the template and an overestimation in mice with lower plaque burden. Interestingly SUVR_CTX∕WM_ and SUVR_CTX∕BST_ suffered a lower bias (<2% relative to the deviation of the individual Aβ-PET image to its template) when compared to SUVR_CTX∕CBL_ and SUVR_CTX∕GLM_ (>10% relative to the deviation of the individual Aβ-PET to its template), supporting the preferred use of the WM and BST reference regions (Figure 3), with matching of stage/age. Furthermore, transient smoothing of the input image with a 0.6 mm filter proved indispensable to avoid strong distortions in the automated spatial normalization.

### Intensity scaling methods

Variance of plain SUV_CTX_ was rather high in BL and FU groups of this investigation (15.9/13.2%), thus indicating inadequate intensity scaling of Aβ-PET images by this method. Well-known sources of error in the SUV_CTX_ calculation include paravenous leakage of the injectate, variability in brain perfusion, and imprecise measurement of radiotracer dosage. We typically calculate injected dose by well-counting syringes before and after tracer application, whereas measurement of the injected dose in a whole body VOI can be more precise (Rominger et al., 2013). However, our scanning configuration does not always capture the entire body of the mice. While SUV measurements are improved with arterial blood sampling, this is inherently linked with a high logistic and economic effort, scarcely feasible in a longitudinal setting. Our SUV_CTX_ findings were distinctly inferior to the several SUVR_CTX∕REF_ results, with respect both to longitudinal and histological validation analyses. Thus, SUV methods are not to be recommended for preclinical Aβ-PET studies.

Having ascertained the need to make SUVR calculations, we need to identify the optimal reference region for intensity scaling. Since a whole brain VOI is easily obtained, scaling to the global mean brain uptake is convenient. Indeed we find a high correlation between SUVR_CTX∕GLM_ and the histological gold standard of Aβ accumulation. However, when large parts of the brain are affected by the amyloid pathology, the denominator for scaling is of course raised by the high tracer retention. This resulted in the spurious detection of declining relative FBB uptake in the hindbrain to follow-up at 6 weeks, an artifact that could mimic a real clearance of fibrillar Aβ in longitudinal interventional designs. Furthermore, global mean scaling attenuated the apparent cluster size and effect size for increases in Aβ in the longitudinal part of the study, relative to findings with scaling to reference regions expected to be devoid of Aβ pathology. Our findings are supported by earlier [^18^F]-FDG-PET investigations of metabolic rate in human brain, in relation to artifactual findings arising as a consequence of global normalization (Borghammer et al., 2009). Scaling to [^18^F]-FDG uptake in a reference cluster unaffected by pathology clearly improved the detection of zones of true hypometabolism in patients with AD or fronto-temporal dementia (Yakushev et al., 2009; Dukart et al., 2013). Thus, we are confident that scaling by reference regions devoid of plaque pathology should perform best for detection of Aβ in the present mouse study.

In most transgenic mouse models, the hindbrain remains relatively unaffected by Aβ plaque accumulation (Teipel et al., 2011), but due to the characteristically high retention of [^18^F]-labeled Aβ tracers in white matter, the binding in hindbrain regions is quite heterogeneous. With regard to FBB-PET, we see low non-specific binding in the cerebellum (Rominger et al., 2013), which led to our adoption of cerebellum as a reference region in small animal Aβ-PET analysis for this and related ligands (Manook et al., 2012). While the cerebellum can serve as an accurate reference region, it remains vulnerable to spill-in from the overlying cranium and adjacent vascular structures (Mille et al., 2012). In addition, cerebellum VOIs are at risk for contamination from non-cerebral voxels, if the caudal border of the brain is not captured precisely. Together, these factors probably account for instances of false high and false low SUVR_CTX∕CBL_; resultant higher variance especially in longitudinal analyses of SUVR_CTX∕CBL_ are responsible for the lower correlation between BL and FU in the present longitudinal arm, as compared to the more precise findings with SUVR_CTX∕WM_ or SUVR_CTX∕BST_. While spill-over and imperfect capture of the caudal brain edges are less of an issue for human Aβ-PET, WM reference scaling seems to give more stable results to follow-up than does cerebellum scaling (Chen et al., 2015; Brendel et al., 2015a). Similarly, the highest effect size (*d* = 1.64) in the present 6-week longitudinal setting was observed for SUVR_CTX∕WM_ and SUVR_CTX∕BST_.

The whole point of Aβ-PET is to depict accurately the plaque load. We designed the present study to test the limits of sensitivity of the method, by examining reference tissue methods in a cohort of PS2APP mice with rather narrow inter-individual range in plaque load (8.6–13.9%). This is in comparison to the ten-fold range used in previous mouse studies of the correlation between Aβ-PET and histological plaque load (1.0–9.2%, 0.3–13.3%; Manook et al., 2012; Rominger et al., 2013). Results with all intracerebral reference regions correlated highly with the histological gold standard, although WM normalization gave a slightly higher correlation. The slight superiority of SUVR_CTX∕WM_ over SUVR_CTX∕BST_ can be explained by the larger volume, giving less statistical noise in the results. The slight superiority of SUVR_CTX∕WM_ over SUVR_CTX∕CBL_, attributed above to imperfect delineation of the caudal limit of the brain, is also demonstrated by the lower correlation of SUVR_CTX∕CBL_ by the novice reader with histology (*R* = 0.66); visual interpretation of caudal limit of the fusion images is a matter of skill-learning.

In summary, SUVR_CTX∕WM_ performed best for the detection of Aβ by PET in transgenic mice, which should prove advantageous in preclinical imaging studies of longitudinal design (Table 4). Thus, quantitation of [^18^F]-florbetaben uptake in interventional trials, where accurate monitoring in single animals is mandatory, can be improved implementing hindbrain white matter intensity scaling, in conjunction with automated spatial normalization.

### Limitations

As mentioned above, we did not use the best possible means to measure the injected FBB dose; we suppose that whole mouse VOIs would have propagated to better correlations of SUV_CTX_ with the histological gold standard. While our PS2APP mice are characterized by almost complete absence of Aβ pathology in the hindbrain, present findings are not translatable to mouse models, which may express relevant Aβ plaque burden in the reference regions tested in this paper. Thus, prior knowledge about the regional deposition of Aβ is indispensable for appropriate selection of the reference in preclinical Aβ-PET studies with FBB or related tracers.

Excessive reliance on automated methods for spatial normalization of mouse brain should be avoided. Although we did not detect any failures of the method, they might still occur, were artificial hot spots present in the image. Thus, we saw distinctly more cases of image distortions, especially in brains with more heterogenous FBB uptake, in the absence of transient input smoothing. As in all PET studies, it is important to visually control post-processed images for potentially failed spatial normalizations. In consideration of this issue, we first attempted automated brain normalization with larger templates (data not shown) encompasing adjacent extracerebral regions, so as to support a correction of effects of partial volume (Brendel et al., 2014). This approach yielded some excessive distortions of the brain, when the VOI extended to include sources in the neck or spine. We note that templates fulfilling the differing requirements for partial volume effect correction and automated brain normalization remain to be validated.

## Conclusion

Automated spatial normalization of mouse brain can be applied to Aβ-PET studies with FBB, thus ensuring pseudo-blinding, and by eliminating inter-reader variability due to differing skill learning. This improves the reproducibility of the endpoint, even if the reader is poorly trained. Intracerebral reference regions lacking Aβ pathology are necessary for accurate *in vivo* quantification of Aβ-PET with FBB; use of a hindbrain WM reference tissue gave the best performance predominately due to advantages in longitudinal designs. SUV_CTX_ generation and global mean scaling were distinctly inferior in performance to the intensity scaling by pathology-free intracerebral reference regions.

## Author contributions

FO carried out the PET experiments, performed the data analysis, and drafted the manuscript; MB participated in the design of the study, contributed to global data analysis, performed the statistical analysis, and drafted the manuscript; AJ carried out the histological experiments, performed the histological data analysis, and drafted the manuscript; VK carried out the histological experiments, and performed the histological data analysis; AD performed statistical programming and analysis; FP carried out the PET experiments, and performed the PET data analysis; CF carried out the PET experiments, and performed the PET data analysis; FG carried out the radiochemistry; JC participated in the PET experiments and helped to draft the manuscript; KB participated in the design of the study and helped to draft the manuscript; CH participated in the design of the study and increased the intellectual content; PB participated in the design of the study and helped to draft the manuscript; JH conceived of the study, and participated in its design and coordination and drafted the manuscript; AR conceived of the study, and participated in its design and coordination, contributed to interpretation of the data and drafted the manuscript. All authors read and approved the final manuscript.

## Funding

The study was financially supported by the SyNergy Cluster (Core 2 project). AJ was supported by the Foundation for Polish Science within the International PhD Project “Studies of nucleic acids and proteins—from basic to applied research,” co-financed by European Union—Regional Development Fund (MPD/2009-3/2).

### Conflict of interest statement

KB is an employee of F. Hoffmann-La Roche; PB received consultant fees from GE and Piramal Imaging, and honoraria from Siemens; AR received consultant fees from Piramal Imaging and GE. The other authors declare that the research was conducted in the absence of any commercial or financial relationships that could be construed as a potential conflict of interest.
